# Process Evaluation of MAPS: A Highly Tailored Digital Intervention to Support Medication Adherence in Primary Care Setting

**DOI:** 10.3389/fpubh.2021.806168

**Published:** 2021-12-20

**Authors:** Aikaterini Kassavou, Charlotte A. Court, Venus Mirzaei, James Brimicombe, Simon Edwards, Stephen Sutton

**Affiliations:** ^1^Department of Public Health and Primary Care, Primary Care Unit, University of Cambridge, Cambridge, United Kingdom; ^2^University Information Services, University of Cambridge, Cambridge, United Kingdom

**Keywords:** medication adherence, process evaluation, behaviour change, Hypertension, Type 2 Diabetes

## Abstract

**Background:** Medication adherence can prevent health risks, but many patients do not adhere to their prescribed treatment. Our recent trial found that a digital intervention was effective at improving medication adherence in non-adherent patients with Hypertension or Type 2 Diabetes; but we do not know how it brought about behavioural changes. This research is a post-trial process evaluation of the mechanism by which the intervention achieved its intended effects.

**Methods:** A mixed methods design with quantitative and qualitative evidence synthesis was employed. Data was generated by two studies. Study 1 used questionnaires to measure the underlying mechanisms of and the medication adherence behaviour, and digital logfiles to objectively capture intervention effects on the process of behaviour change. Multilevel regression analysis on 57 complete intervention group cases tested the effects of the intervention at modifying the mechanism of behaviour change and in turn at improving medication adherence. Study 2 used in depth interviews with a subsample of 20 intervention patients, and eight practise nurses. Thematic analysis provided evidence about the overarching intervention functions and recommendations to improve intervention reach and impact in primary care.

**Results:** Study 1 found that intervention effectiveness was significantly associated with positive changes in the underlying mechanisms of behaviour change (*R*^2^ = 0.26, *SE* = 0.98, *P* = 0.00); and this effect was heightened twofold when the tailored intervention content and reporting on medication taking (*R*^2^ = 0.59, *SE* = 0.74, *P* = 0.00) was interested into the regression model. Study 2 suggested that the intervention supported motivation and ability to adherence, although clinically meaningful effects would require very brief medication adherence risk appraisal and signposting to ongoing digitally delivered behavioural support during clinical consultations.

**Conclusion:** This post trial process evaluation used objective methods to capture the intervention effect on the mechanisms of behaviour change to explain intervention effectiveness, and subjective accounts to explore the circumstances under which these effects were achieved. The results of this process evaluation will inform a large scale randomised controlled trial in primary care.

## Introduction

Medication adherence can prevent morbidity and mortality associated with Hypertension and Type 2 Diabetes (1, 2). However, many patients do not adhere to their prescribed treatment (3), contributing to increased cost for the National Health Service (4). Currently there are no effective ways to improve adherence (5) and the UK Department of Health recommends that cost-effective and innovative interventions should be developed and evaluated.

We have therefore developed a highly tailored and interactive behavioural intervention, the Medication Adherence for Patients Support (MAPS) to support treatment adherence in primary care. To develop the intervention, we have reviewed theory, evidence, and obtained insights from Patients and Public Involvement and Engagement (6–9).

The MAPS intervention aims to support medication adherence by modifying the theoretical determinants that underpin behaviour change; that is, the non-intentional and intentional non-adherence, the health outcome expectations, the medication adherence self-efficacy and the social norms. One way to effectively modify the underlying mechanisms of behaviour change is to provide highly tailored advice to the individual and further support reports on behavioural performance (8). The intervention utilised behaviour change techniques (10) and strategies (8) aiming at modifying each or combination of the theoretical determinants and thus to bring about change in medication adherence behaviour (6).

The MAPS intervention was evaluated at a pragmatic randomised controlled trial with 135 non-adherent patients with Hypertension or Type 2 Diabetes recruited from eight primary care practises in the UK; and it was found to be effective at improving medication adherence and reducing blood pressure and glucose levels (11). However, we do not know how the intervention brought about changes on medication adherence behaviour (12, 13).

Disentangling the effective and replicable intervention processes that bring about improvements in medication adherence could inform effective and scalable medication adherence programmes in primary care. Though, to date our knowledge about the ways by which medication adherence is improved is based on self-reports, which makes the identification of effective and replicable interventions challenging.

This research is a post-trial process evaluation of the MAPS intervention which utilised the technology to collect objective evidence to inform knowledge about the ways by which the intervention achieved its indented effects. It has also obtained subjective accounts to explore the implementation conditions that enabled intervention reach and impact in primary care setting. The ultimate aim of this research was to inform evidence for effective and replicable medication adherence interventions real-world practise (13).

## Materials and Methods

### Description of the MAPS Intervention

The Medication Adherence for Patients Support (MAPS) is a highly tailored and interactive behaviour change intervention: the content of the intervention was tailored to each patient's values of intentional non-adherence, non-intentional non-adherence, health outcome expectations, medication adherence self-efficacy and social norms. The content, intensity and schedule of the intervention messages was pre-specified based on theory, qualitative and quantitative evidence, consultations, and Involvement and Engagement of the Public (6). The digital mode to facilitate the behaviour change intervention was interactive voice and text messaging. The duration of the intervention was three months.

The intervention included three broader categories of messages: (1) advice messages tailored to the theoretical determinants of medication adherence, (2) reminder messages about the prescribed regimen, and (3) query messages that included behaviour change strategies to prompt active engagement with the tailored intervention content by asking patients to report whether or not they have taken their medications as prescribed (for example of messages, see Supplementary Table 1). Participants had the option to reply to these queries in real time during the pre-scheduled automated phone calls, or at any time using the inbound function of the interactive voice response or the text messaging service.

The schedule (i.e., sequence of messages) and intensity (i.e., target of one or more theoretical determinants) of the intervention was pre-specified as following: during the first week, each patient was asked to complete questions about the determinants of medication adherence behaviour and was sent highly tailored feedback aiming to address these behavioural determinants. During the first month, the intervention included more reminder messages, which were gradually reduced during the second month and were stopped during the third month of the intervention. Advice messages followed a reverse delivery sequence: they were introduced during the first month and gradually replaced the reminder messages during the second and the third intervention month.

We adopted a flexible approach to intervention delivery: the number of the reminder messages and the timing of all messages were pre-selected by participants, and participants could change these options during the 3-month intervention i.e., they had the option to request less or more messages, or to stop receiving messages.

### Recruitment and Setting

The trial was implemented in eight primary care practises in the UK. Recruited primary care practises were located at different areas of deprivation, with the majority of them in deprived or highly deprived areas, ensuring that the trial reached patients from a wide range of deprivation areas. Practise nurses who advised patients about medication adherence, blood pressure checks or other similar consultations, were invited and participated in the trial.

Patients were eligible to participate if they met all four inclusion criteria: (1) were above 18 years old, (2) had a diagnosis of either Hypertension or Type 2 Diabetes mellitus, or both health conditions; (3) had been prescribed at least one antihypertensive medication or glucose lowering medication; and (4) had either poorly controlled blood pressure or glucose levels as logged in their medical records, or had gaps in collecting repeat prescriptions during the six months before study invitation. Patients were excluded if they were taking part in another medication adherence intervention or had a health condition that could impair their participation.

Eligible patients were identified from the practise database by a practise manager and confirmed for eligibility by a practise GP. Patients were then approached opportunistically by practise nurses during usual care consultations or proactively by sending text message or postal invitations. Patients were prompted to contact their practise nurse or the research team, to book their baseline consultation. During baseline consultation patients provided written informed consent and completed baseline data measurements. All methods and procedures have been approved and were carried out in accordance with the guidelines and regulations of the Ethics Committee of East of England, Essex Research Ethics Committee (REC Reference number 17/EE/0203) and Health Research Authority.

At completion of baseline measurements, patients were randomised to either the digital intervention as an adjunct to usual care or to usual care only. The random sequence was generated by a centralised web-based service and was stratified by two important confounders: medication adherence intention as measured by the Medication Adherence Report Scale (14) and burden of pills. MARS threshold of 24 was selected to indicate low (below 24) or high (above or equal to 24) intention to adherence. Burden of pills ratio of 10:6 (10 tablets: six different health conditions) was selected to indicate low (below 10:6) or high (above or equal to 10:6) burden of pills; and ratio was based on our pilot studies (6). The data to calculate the burden of pills was extracted by objective records of patient most recent repeat prescription recorded in practise databases. More information about the trial design and implementation procedures is provided elsewhere (11).

### Process Evaluation

A mixed methodology was employed in line with recommendations for the process evaluation of randomised controlled trials (12, 13). In line with this, we synthesised data generated by two supplementary studies: Study 1 was a quantitative evaluation of the mechanisms of behaviour change and therefore of intervention effectiveness; and Study 2 was a qualitative evaluation of the circumstances under which these mechanisms brought about medication adherence in non-adherent patients with Hypertension or Type 2 Diabetes in primary care.

We collected data, using questionnaires, digital log files and in-depth interviews, to triangulate data synthesis and generate findings.

The population for this process evaluation was (1) the intervention group patients only, selected due to the data utilised for the process evaluation i.e., digital log files to objectively capture intervention effects, and (2) both intervention group patients and health care facilitators (i.e., practise nurse), given the importance of this evidence to inform future medication adherence programs in primary care.

Specifically, the process evaluation aimed to respond to the following research questions:

a) Was intervention effectiveness associated with improvements in the theoretical determinants that underpin behaviour change i.e., intentional and non-intentional non-adherence, medication adherence self-efficacy, health outcome expectations and social norms?
b) Did tailoring and reports on behaviour moderated the effects of the theoretical underpinnings at improving medication adherence?
c) What were the overarching intervention functions that supported intervention effectiveness?
d) What were the conditions under which the intervention achieved reach and impact of non-adherent patients in primary care setting?

Study 1 provided evidence to respond to research question a and b; and study 2 provided evidence to respond to research questions c and d.

### Data Collection and Coding

#### Study 1. Quantitative Evaluation

To evaluate the underlying mechanisms of behaviour change we collected data using a baseline and 3-month follow up questionnaire measuring non-intentional non-adherence, intentional non-adherence, health outcome expectancies, medication adherence self-efficacy, social norms and medication adherence behaviour.

Non-intentional non-adherence refers to patients' non-conscious consideration of performing a behaviour; such as forgetting or misunderstanding the prescribed treatment. Intentional non-adherence refers to patients' conscious consideration of performing a behaviour; such as not taking their medications as prescribed because they decide not to take a dose or stop taking their medications (15, 16). Non-intentional non-adherence and intentional non-adherence were measured using the MARS (14): one item measured non-intentional non-adherence (‘I forget to take my tablets’), and four items measured intentional non-adherence (4-items, Cronbach's α = 0.924 e.g., “I alter the dose of my tablets,” “I stop taking my medications for a while”).

Health outcome expectations refers to patients' outcome expectations, such as the perceived reduction in risks of developing health complications that follows medication adherence (17). Health outcome expectations was measured by a single item (“If I were to take my meds as prescribed and without missing a day it would reduce my chances of developing complications from the health condition I've been diagnosed with”).

Medication adherence self-efficacy refers to patients' perceptions about their ability to take all the doses of their prescribed medications as prescribed, as well as their optimistic beliefs about their ability to sustain their behaviour regardless the barriers specific to long-term medication adherence (17). Such barrier may include the perceived burden of pills (e.g., perception about the number of pills and complexity of health condition) or the focus on the emotional state (e.g., emotional state as a primary drive of the behavioural performance). Medication adherence self-efficacy was measured by three single items; one item measuring generic medication adherence self-efficacy (“I am confident that I can take all my prescribed tablets without missing a day”), and two single items measuring self-efficacy to long-term adherence (ability to sustain adherent regardless the perceived burden of pills “I am confident that I can take all my medication as prescribed every day and without missing a day, even if I have other medications to take”; and ability to sustain adherent regardless the emotional state “I am confident that I can take all my medications as prescribed every day and without missing a day, even if I am stressed out”). The last two items were selected by the Medication Adherence Self Efficacy Scale questionnaire (18), and the decision on selecting these two items was based on the results of our previous studies that have identified the barriers to medication adherence (6, 7).

Social or subjective norms refer to perceptions about others' views about taking medication or others' adherence to medication (e.g., beliefs about others medication adherence behaviour) (16). Social norms were measured by two items (Cronbach's α = 0.800 “most people who are important to me would approve of me taking all my prescribed tablets without missing a day,” “if they were prescribed tablets, most people who are important to me would take all their prescribed tablets without missing a day”).

Medication adherence was measured by one single item (“how many days in the past week have you taken all your prescribed tablets?”).

To disentangle the intervention content that brought about change, we coded the data objectively captured by digital log files during the three months intervention based on the following operational definitions: we coded the variable “tailoring” when there was a confirmation of receipt of the tailored intervention advice. The median score for tailoring was used as a threshold to indicate low (below or equal to threshold) or high (above threshold) tailoring.

We coded the variable “report behaviour” when there was a “yes” or “no” response to intervention query messages to report medication taking. Report behaviour was coded as “high” when all responses confirmed medication taking, “medium” when at least half of the responses confirmed medication taking and “low” when less than half of the responses confirmed medication taking.

Tailoring and report on behaviour were conceptualised and coded as different variables, because tailoring required confirmation of the tailored intervention advice, whereas report on behaviour required confirmation of the behavioural performance (8).

We have also coded “overall intervention usage,” that is a combined score of the objectively measured intervention components received i.e., confirmation of receipt of the tailored intervention content, responses to intervention query messages, and interactions regarding the intervention delivery options (e.g., request to receive more or less messages)—to explore potential effects of the overall intervention usage on intervention effectiveness. Data that captured usage of the intervention regarding the study procedure (i.e., messages about completing study visits or procedures) were excluded.

Data captured at digital log files during the three months intervention were extracted and coded. Each patient's digital log files were coded separately. Data summarising tailoring, report on behaviour and overall intervention usage across all participants was then grouped into one coding and included in the analysis.

#### Study 2. Qualitative Evaluation

The qualitative evaluation explored two aspects of intervention impact; (a) the overarching intervention functions that supported intervention effectiveness; and (b) the conditions under which the intervention reached non-adherent patients with Hypertension or Type 2 Diabetes in primary care.

We conducted in-depth interviews with a subsample of the intervention group patients and all practise nurses or health care assistants who took part in the trial, to obtain multi-perspective views about the individual and context-specific elements of the intervention reach and effectiveness.

The patients semi-structured interview guide was developed by a researcher based on theory and aimed to explore views about the intervention content and prompt recommendations for improvement. Patients were asked their views about specific intervention messages, whether and how messages supported medication adherence and under what circumstances. When patients could not remember or elaborate about a specific intervention message, an example of a received message as recorded by digital log files was provided.

Each of the patient's interviews lasted from 90 to 180 min. Longer interviews with patients were required to establish rapport and overcome potential bias regarding the role of the interviewee and the interviewer. The first 13 interviews were conducted face-to-face at patient's home until main codes were created, the remaining seven interviews were conducted by phone to confirm or further explore the elicited codes.

The semi-structured interviews with practise nurses aimed to explore elements that impacted on intervention reach and obtain recommendation to improve intervention scalability in primary care. Practise nurses were asked about patients' characteristics for whom the intervention was acceptable and potentially effective, and the practise-level conditions that could facilitate intervention reach and scale up (for a copy of the interview guides, see Supplementary File, interview schedule). Practise nurse interviews took place at the completion of patients' recruitment, were conducted face-to-face at the GP practises, and each interview lasted for an average of 45 min.

Three members of the research team (AK, CAC and VM) conducted the interviews independently. AK conducted the interviews with practise nurses, AK and VM conducted the face-to-face interviews with patients, and CAC and VM conducted the telephone interviews with the patients. All interviews were audio-recorded and transcribed by an independent transcription service. Transcripts were double checked for accuracy against recordings by the researchers, and all personal identifiable data were removed before analysis. Field notes were collected during the face-to-face interviews to inform data analysis. One of the researcher's had experience in developing and evaluating medication adherence interventions, including process evaluation using quantitative and qualitative methods, and the two others had training on qualitative data collection and analysis. The researchers had no previous knowledge or relationship with the participants.

Questionnaires and interviews were completed by patients at the end of the 3-month intervention; from June 2018 to April 2019. Data were recorded at the digital log files during the 3-month intervention; from March 2018 until March 2019. Quantitative data coding and analysis was conducted during December 2019. Qualitative coding was conducted by two researchers, and analysis were conducted during September 2019. Data triangulation and mixed methods analysis was completed during January 2020. The trial was first registered at ISRCTN on 08/08/2017 and the reference number is 10668149.

### Sample Size

#### Study 1

In total 77 patients were randomised and enrolled into the intervention group, and 57 of them provided complete data and included in the quantitative analysis.

#### Study 2

The selection of patients who were invited to take part at a post-trial in depth interview was informed by the coding of the digital log files: invited participants were selected based on level of intervention engagement (high engagement: daily use of the intervention for more than 11 days; or low engagement: ≤11 days) and basic demographics (e.g., age, gender, deprivation level) to ensure that a variety of views were explored. From those meeting the eligibility criteria, we randomly selected 25 and invited them to take part in the interview using phone calls. Five participants refused to participate, three because of time constraints and two because of lack of interest. In total 20 patients completed the end of intervention interview: 13 face-to-face and seven over the phone.

Eight practise nurses, one from each of the eight primary care practises, who either identified or invited patients during usual care consultations, were invited by phone and took part in the in-depth face to face interview.

### Data Analysis

#### Study 1. Quantitative Evaluation

Principal component analysis suggested that multi-collinearity was not an issue for the variables. Histograms explored continuous variables' distribution, and the Levene test assessed the assumption of equality of variance. Regression analysis explored whether intervention effectiveness was associated with the underlying mechanisms of behaviour change. Multivariable regression analysis tested whether, and to what extent, intervention tailoring and report on behaviour modified the effects of the theoretical underpinnings at improving medication adherence. Data were inserted into the regression model, with medication adherence as a dependent variable and each of the theoretical determinants as an independent variable. Interactive effects were explored between the theoretical determinants with tailoring and report on behaviour. The variables measuring theoretical determinants were adjusted for baseline values. Analysis was conducted using SPSS v26.

#### Study 2. Qualitative Evaluation

Qualitative data were analysed thematically (19) using NVivo. At the first stage of the qualitative analysis the researchers worked independently: they coded each transcript using an inductive approach and developed one mind map for each of the transcripts. During the second stage of the analysis, the researchers met and discussed each transcript and mind maps and merged themes and sub-themes into one mind map. At the third stage of analysis, the researchers met and merged all mind maps into a broader mind map describing the main themes and subthemes, using a deductive approach. Analysis was completed when data saturation was achieved. Any additional data generated by the inductive approach were treated as recommendations for improvements.

## Results

The majority of patients were registered with primary care practises located at highly deprived areas and were above the age of 50 years. The 3-months follow up results on behavioural and clinical outcomes have been reported previously (11). Medication adherence was significantly improved in the intervention group compared to control (improvement of 2 days, *P* = 0.04; 6.85 ± 0.47 vs. 6.36 ± 1.59). Similar direction of effects was observed for improvements at both the systolic blood pressure (reduction of 0.6 mmHg, 95%CI −7.423 to 6.301) and glucose levels (reduction of 4.53 mmol/l, 95% CI −13.099 to 4.710) favouring the intervention group.

### Study 1. Quantitative Process Evaluation

At 3 months, improvements in medication adherence were positively and significantly associated with improvements in intentional non-adherence (*b* = 0.46, *P* = 0.03), non-intentional non-adherence (*b* = 0.77, *P* = 0.00) and medication adherence self-efficacy (*b* = 0.34, *P* = 0.00) within the intervention group. There was a trend towards positive associations between medication adherence and improvement on each of the health outcome expectations (*b* = 0.23, *P* = 0.08) and the two specific self-efficacy variables (*b* = 0.32, *P* = 0.07 for burden of pills; *b* = 0.14, *P* = 0.24 for emotional state), but these effects were not statistically significant. There were no effects of the social norms on intervention effectiveness (see Supplementary Table 2; Supplementary Figures 1–4).

During the 3 months intervention, objective measures confirmed high intervention tailoring (73.4%, 42/57), which was significantly associated with improvements in medication adherence (*b* = 1.02, *P* = 0.01). Reports on medication adherence was 26.8% low, 60.7% medium and 12.5% high, and it was positively and significantly associated (*b* = 1.32, *P* = 0.00) with improvements in medication adherence within the intervention group (see Supplementary Figures 5, 6). There were no effects of overall intervention usage at explaining intervention effectiveness.

Multilevel regression analysis suggested that intervention effectiveness was explained by positive changes in intentional non-adherence, non-intentional non-adherence and medication adherence self-efficacy (*R*^2^ = 0.26, *SE* = 0.98, *P* = 0.00), and this effect was heightened further when tailoring (*R*^2^ = 0.32, *SE* = 0.95, *P* = 0.00) and report medication adherence behaviour (*R*^2^ = 0.59, *SE* = 0.74, *P* = 0.00) was included in the regression model (see Table 1).

**Table 1:** Model summary.

**Model**	**R**	**R square**	**Adjusted R Square**	**SE**	**F change**	**Sig. F Change**
1	0.51^a^	0.26	0.21	0.98	6.00	0.00
2	0.56^b^	0.32	0.26	0.95	6.11	0.00
3	0.77^c^	0.59	0.55	0.74	14.59	0.00

a *Predictors: intentional non-adherence, non-intentional non-adherence, medication adherence self-efficacy*.

b *Predictors: intentional non-adherence, non-intentional non-adherence, medication adherence self-efficacy, tailoring*.

c *Predictors: intentional non-adherence, non-intentional non-adherence, medication adherence self-efficacy, tailoring, report behaviour*.

*Variables intentional non-adherence, non-intentional non-adherence and medication adherence self-efficacy are adjusted for baseline values*.

### Study 2. Qualitative Process Evaluation

The qualitative analysis obtained subjective accounts about the overarching intervention functions and obtained recommendations to improve intervention effectiveness, reach and scale up in primary care.

Three overarching themes were identified: the intervention (a) facilitated motivation to medication adherence; (b) enabled medication adherence behaviour, and (c) prompted social integration. To improve intervention impact and scale up, participants recommended the integration of the behavioural intervention into usual care consultations to facilitate control over the long-term clinical indicators of the health condition.

#### Intervention Facilitated Sustained Motivation to Adherence

Patients reported that their motivation to take medication was embedded in improving their health condition and thus achieve health benefits, whereas barriers to adherence were mainly influenced by their everyday lifestyle; and that the intervention supported them to sustain adherent by reinforcing their motivation and by prompting them to specify and address the barriers to adherence (see Table 2, quotes 1.1).

**Table 2:** Qualitative data.

	**Quotes per theme**
**1**	**Intervention facilitated motivation to adherence**
1.1.	“(I do not take my tablets) in the morning, if I break the routine, and start doing something else, when I don't follow my routine” patient 10042 “it (the intervention) emphasised … the importance of you not forgetting to take medication and the effect that it could have if you didn't take it. So, I think it was quite useful … they weren't very detailed message so, it was quite short message, about taking your pills …it gave you a bit of a jolt, so you did not become complacent about what it is, and stress I should be taking it” patient 50012 “I felt mainly motivation from it” patient 30006 “That me saying yes, my name is John you know, and then they gave me a good, nice message, and it was always relevant” patient 20031
1.2	“And make sure you take them exactly at the same time all the time cos I used to vary my times in taking the tablets… a few minutes it does not matter either way, but if, you know, you don't get the text you might not take them, you might forget all about them” patient 10003 “I suppose it made sure that I took my tablets, which I normally do anyway, but it made me take them at a more specific time” patient 50012 “Okay, well, for me it (the intervention) was positive, it definitely helped me get into the habit more of my medication because I don't really have a routine, so because of that, I'm here, there, everywhere doing things, cos I don't, cos I don't have a routine” patient 10042 “since the phone calls, I do not know whether it's subconscious and “make sure you take them exactly the same time, all the time” patient 10038
1.3	“Yeah … this is, erm, what the tablets are gonna do for you in a positive manner going forwards, you know, maybe not, not tomorrow or next week, but this time next year” patient 10042 “but if you get a reminder it makes you think perhaps it is important (to take your medications)… so I think, when somebody's prescribed a new medication, send them message for couple of months, two or three months, to establish a pattern” 50013
**2**	**Intervention facilitated patients' sustained ability to adherence**
2.1.	“Maybe I should have had it at 09:00, maybe that's when I should have asked for the reminder” patient 50013 “it's, you know, it's just sort of being at right place, right time, no interruptions” patient 50012 “it makes you more of your condition and how it should be… it just wanted to be certain people were actually getting the responsibility to it” patient 50012 “Maybe at the midpoint (of the intervention), because people might not know what their triggers are early on … just have a quick review what's working best and then perhaps tailor things slightly more to that person once they've had the experience of the service that comes through … after a months or two months … there is the opportunity to tailor it to that person” 30006 “well basically just made me think about it and think how I could actually work to control it in a particular way … I've forgotten, and you know, I sort of tried to take on board what the whole system was and try to help myself in the process” patient 10043
2.2.	“mind you, medications don't always make you feel better, do they? … you ask people what they thought, and you get a blatant truth about how they thought and felt, and I said, oh … so, you've got to be careful what you ask” patient 50012
2.3.	“I think it is important that people know that if they feel that it's not beneficial or it's making them feel ill that they should go back to their GP and chat to them about it” patient 10042 “it made clear that, if I am feeling good it's because I' m doing that (taking my medications)” patient 20031
2.4.	"I would basically recommend more messages about the behaviour and taking or not taking tablets rather than how you feel about taking or not taking tablets?” patient 50012
2.5.	“It was a random selection of what the message was going to be, you know, it just felt a bit more personal … I could relate to that, just because the messages were different, it was varied, and you felt it … there was somebody there talking, and you know, it was more geared to you and your medication … that is something that will set you on the path to take your medication as prescribed” patient 50012
2.6	“On a personal level, ask people “did you take your medication?” and then send a text message saying “according to you, you took 85% of your medication this month, that' good, but you can improve” … and that'll just help people think “oh blimey, is that all I've done?”…just make people aware of how well they really are doing” patient 20031
**3**	**Intervention prompted integration of medication adherence in the social context**
3.1.	“I've told my grandchildren and let them listen to the messages … and they were copying it… but everyone knows that (I am taking medications), you know, and will say to me, “have you taken your tablets?”, especially if we're out for lunchtime … and my husband at 09:00 he is saying ‘go and take your tablets’, I say “I'll go in half an hour,” and he is like “no, you'd better take them now” … and my daughter said to me the other say “who are you taking to” and I said “Its MAPS calling about my pill reminder” and she is “oh yeah, that's okay” patient 50012 “I liked the message to remind you to ask your husband or a friend to remind you, that was good … I have not really thought about this, because it is a private thing, isn't it” patient 50013
3.2.	“it's (the intervention) speaking to you basically instead of just a sentence coming up on your mobile phone… I think a lot of people, if they live on their own and they are a bit lonely or whatever, it's a voice at the other end of the phone” patient 10038 “I suppose the voice messages had more of an impact because you had to take note of them more and listen and it made you think a bit more than the text messages, because they were just simple” patient 20052
3.3.	“It is like someone is paying notice on how you are, and how are you doing with (taking) your medication in everyday life” patient 20031 “I think they like the idea that it is offered by a practise nurse…they feel this extra support” practise nurse 10025
3.4.	“you have less chance especially in a big family of them (the intervention messages) going to the right person, so, it does help just sort of like do it on their own personal mobile phone… there was no privacy attached to the intervention messages, and taking medication is a private thing” patient 10036
3.5.	“Because people say “oh waste of money, do not take them, they're not gonna do any good. I've taken them for so long, they haven't done me any good” … then I thought I am not gonna take notice of anybody else, I'm going to start doing this (taking my tablets) on a regular basis… it's important that you take these things because you've got to look after your health” patient 10045
**4**	**Recommendations to improve intervention efficacy, as well as reach and scale up in primary care**
4.1.	“it is challenging to recruit patients who do not even attend their practise appointments, am not sure how you could convince these people, we tried several times but with no response” practise nurse 10030 “[when we followed up the invitation to the study] they did not feel there was a problem and that was, that was the, the answer mainly we got back from them, “no there's no problem, I'm taking the medication and I do not forget” so even though their clinical signs were that their blood pressure was out of control or their blood sugars were raised, they said they took their medication” practise nurse 10025 “with the searches you can see when they last had one (prescription), it is recorded, but there is a small core of participants that will deny that there is a problem whatever you put in front of them” practise nurse 10026 “some of these, they do not want to take the amount of tablets, they want to take less” practise nurse 10032 “If the practise feels there was a problem with adherence, so we should refer patients to additional support to help solve the problem” practise nurse 10031
4.2.	“identify them when we see their blood results, perhaps on their annual review … and ask them if they would like to take up this service… and probably do not ask them if they take their tablets, when we asked them, they always say “yes” practise nurse 10026 “there are some people who need evidence that this service will work for them” practise nurse 10032
4.3.	“in theory there should be enough time for a very brief intervention within the consultation, but sometimes we get extra patients added in so then you've got to, you know, if you get emergency patients coming in or whatever, then you do not even get your 20 min so then it would be very difficult” practise nurse 10027 “Perhaps a small appointment time which is taken up by the whole of the review” practise nurse 10029 “I think they would participate in the intervention, as long as this it is a shorter questionnaire and it [the intervention] is recommended by their practise nurse…a very brief introduction about the clinical signs and response to adherence and then the text message” practise nurse 10026
4.4.	“probably if there was a mechanism for the patient to report back to their health care provider about their health and well-being” practise nurse 10028

It was also reported that the intervention facilitated awareness of medication adherence and reduced the perceived complexity of the prescribed regimen i.e., burden of pills. This was particularly useful for those patients who at baseline reported that they took more tablets than those recorded at their medical records (see Table 2, quotes 1.2). Furthermore, it was reported that the intervention supported daily adherence to medication and the acceptability of adherence to prescribed treatment in the long term (see Table 2, quotes 1.3).

#### Intervention Facilitated Patients' Ability to Adherence

The intervention messages prompted participants to contextualise and adjust their medication taking behaviour to achieve adherence. It was reported that the intervention raised awareness about the circumstances under which the behaviour is performed and enable them to exercise control over and adhere to their prescribe regime (see Table 2, quotes 2.1)

All patients reported that medication adherence is a dynamic process and affective attitudes about medication taking are influenced by side effects and vice versa (see Table 2, quotes 2.2). Thus, it was recommended that the advice about medication adherence affective attitudes should be tailored to patients' emotional state, their available resources and facilitate access to additional support when required.

Some patients reported that the intervention facilitated affective attitudes about medication taking when their emotional state might counter behavioural performance (see Table 2, quotes 2.3). They suggested that the messages to address medication adherence affective attitudes should be linked to behavioural performance and not to generic emotional state; primarily because their affective attitudes are informed by the behaviour (see Table 2, quotes 2.4)

Many patients reported that the tailored intervention content enhanced the acceptability of the advice provided, increased relevance and enabled medication adherence (see Table 2, quote 2.5). Patients recommended to integrate feedback on behavioural performance to further enable sustained medication adherence (see Table 2, quote 2.6).

#### Intervention Prompted Integration of Medication Adherence in the Social Context

The intervention messages prompted integration of medication adherent behaviour in the social context (see Table 2, quotes 3.1), especially for those patients who reported favourable social norms about medication adherence. Patients with favourable social norms but lack of social support reported that the tailored messages provided them with emotional support and prompted generic social integration (see Table 2, quotes 3.2). These patients also reported higher levels of satisfaction with their health care provider and GP practise (see Table 2, quote 3.3).

However, patients with unfavourable social norms about taking medications reported concerns with receiving support for medication adherence (see Table 2, quote 3.4). Nevertheless, some patients reported that the intervention supported medication taking even when social norms or practical social support was not in favour of medication adherence (see Table 2, quote 3.5.).

#### Recommendations to Improve Intervention Reach and Scale Up

Practitioners reported limited ability to address medication non-adherence, highlighted the challenge to engage non-adherent patients on shared decision making about taking and adhering to their prescribed treatment; and they recognised the need to signpost patients to additional behavioural support (see Table 2, quote 4.1). To increase scale up, practitioners recommended to integrate brief risk behavioural appraisal into blood pressure checks and diabetes reviews (see Table 2, quote 4.2) and recommended effective ways and methods to signpost patients to additional digitally delivered behavioural support (see Table 2, quote 4.3), that could be more feasible within the time constrains of primary care consultations and could increase intervention impact and scale up (see Table 2, quote 4.4).

## Discussion

### Principal Findings

This post-trial study evaluated the process by which the MAPS intervention improved medication adherence in non-adherent patients with Hypertension or Type 2 Diabetes in primary care. It was found that the intervention improved medication adherence by modifying the underlying theoretical determinants of intentional non-adherence, non-intentional non-adherence and medication adherence self-efficacy (these explained 26% of intervention effectiveness). Intervention tailored advice and reporting on behaviour captured objectively by digital log files significantly amplified these effects by twofold (heightened explanation of intervention effectiveness at 59%).

Qualitative data supported that the intervention increased motivation to adherence, enable patients to sustain adherent and prompted integration of medication adherence behaviour into social context. To improve intervention reach and scale up, the intervention could provide very brief behavioural risk appraisal during usual care clinical consultations and signpost patients to an ongoing digitally delivered behavioural support.

### Strengths and Limitations

The results of this research were based on data obtained by the intervention group of patients taking part in a pragmatic randomised controlled trial implemented in primary care setting. To our knowledge, this is the first process evaluation of a medication adherence digital intervention in the primary care. This study elucidated the mechanism by which the intervention brought about behaviour change in non-adherent patients and provided the evidence-base of effective medication adherence interventions in primary care.

A strength of this research is the measurement of intervention content captured objectively by digital log files, which provided objective data about patients' engagement with the tailored intervention content. The research has also obtained data from multiple perspectives and facilitated data triangulation. Another strength of this research is its mixed methodology approach. This process evaluation synthesised both quantitative and qualitative evidence to provide comprehensive responses to our research questions.

A limitation of this research is the lack of evaluation of the underpinnings of health behaviour change against the control group of the trial. However, between group comparisons were not possible due to the primary aim of this study and the data required to respond to the research questions. The evidence of this research is based on a subsample of patients, and the results should be treated with caution when interpreted to larger population. Although the sampling was stratified and controlled for important confounders, quantitative process evaluation might require data from larger samples to provide the necessary power to detect the impact of digital interventions on modifying the determinants of medication adherence and on subsequently improving adherence.

This research study did not control for the effect of other potentially important cofounders on the intervention effectiveness, like the effect of the health care provider or patient demographics (e.g., gender, age, ethnicity). Future research could explore the effect of these cofounders on intervention effectiveness, to increase knowledge about the scalability of the intervention to non-adherent patients.

### Implications to Improve Intervention Effectiveness and Impact

This research provided evidence about the process by which a behavioural intervention improved medication adherence. It was found that the intervention effectiveness was supported by improvements in non-intentional non-adherence, intentional non-adherence and medication adherence self-efficacy. This finding suggests that intention to adherence and positive appraisals about the capability to medication adherence are important mechanisms of effective interventions. Furthermore, this study found that the intervention tailored advice and reports on behaviour heightened this effect twofold at improving medication adherence. Positive improvements were observed for the perceived reduction in risks of developing health complications that follows medication adherence and beliefs about ability to adherence regardless the perceived burden of pills, suggesting that these could potentially be effective mechanisms of medication adherence.

Future research could usefully investigate the effects of these mechanisms using larger sample size and against the control group to provide rigorous evidence about the mechanisms of medication adherence. A combination of highly tailored and interactive digital intervention with very brief health care provider behavioural advice about medication adherence could act synergistically to strengthen the impact of the intervention in the primary care.

### Implications for Policy and Practise

Participants actively engaged with the tailored intervention daily for an average of 11 min, during the intervention (duration of daily messages was ~1 min). Considering the challenges to engage non-adherent patients with usual care advice (20), and the limited time of the health care professionals to provide ongoing support, this engagement score suggests that this digital intervention is feasible and could provide brief, effective and real-time support to improve medication adherence in non-adherent patients. It also suggests that this digital intervention could be a cost-effective solution for the provision of health care services.

## Conclusion

To our knowledge this is the first research that has evaluated the mechanisms by which an interactive text and voice message intervention has supported medication adherence within non-adherent patients in the intervention group of a randomised controlled trial in primary care. It was found that tailored intervention content and reports on behavioural performance doubled the effect of intentional non-adherence, non-intentional non-adherence, and adherence self-efficacy in explaining improvements in medication adherence. Patients reported motivation and ability to be important intervention effects in improving medication adherence behaviour. Practise nurses recommended very brief medication adherence risk appraisals followed by signposting to additional digitally delivered behavioural intervention to support non-adherent patients as part of the time constrained usual care consultations. Future research could usefully investigate and evaluate the effects of active and objectively captured intervention content at modifying the mechanisms of behaviour change and at improving medication adherence and clinical outcomes using rigorous designs.

## Data Availability Statement

The raw data supporting the conclusions of this article will be made available by the authors, without undue reservation.

## Ethics Statement

All methods and procedures have been approved and were carried out in accordance with the guidelines and regulations of the Ethics Committee of East of England, Essex Research Ethics Committee (REC Reference number 17/EE/0203) and Health Research Authority. The patients/participants provided their written informed consent to participate in this study.

## Author Contributions

AK and SS have developed the intervention and designed this study. CC and VM assisted with patients' invitation to the telephone interviews, qualitative data collection, and data coding, supervised by AK. JB and SE developed the digital platform to facilitate the delivery of the tailored behavioural intervention and to capture data about patients' intervention usage at digital log files. AK conducted the analyses and drafted this publication, with comments and advice by SS. All authors have read and approved this manuscript for publication.

## Funding

This paper presents independent research funded by the National Institute for Health Research (NIHR) under the Research for Patient Benefit program [grant number PB-PG-0215-36032].

## Author Disclaimer

The views expressed are those of the authors and not necessarily those of the NIHR or the Department of Health and Social Care.

## Conflict of Interest

The authors declare that the research was conducted in the absence of any commercial or financial relationships that could be construed as a potential conflict of interest.

## Publisher's Note

All claims expressed in this article are solely those of the authors and do not necessarily represent those of their affiliated organizations, or those of the publisher, the editors and the reviewers. Any product that may be evaluated in this article, or claim that may be made by its manufacturer, is not guaranteed or endorsed by the publisher.

## Abbreviations

MAPS: Medication Adherence for Patients Support
MARS: Medication Adherence Report Scale.

## References

[1] Kettani F-Z Dragomir A Côté R Roy L Bérard A Blais L. Impact of a better adherence to antihypertensive agents on cerebrovascular disease for primary prevention. Stroke. (2009) 40:213–20. 10.1161/STROKEAHA.108.522193 19038916

[2] Breekveldt-Postma NS Penning-van Beest FJA Siiskonen SJ Falvey H Vincze G Klungel O. The effect of discontinuation of antihypertensives on the risk of acute myocardial infarction and stroke. Curr Med Res Opin. (2008) 24:121–7. 10.1185/030079908X253843 18031596

[3] Mazzaglia G Ambrosioni E Alacqua M Filippi A Sessa E Immordino V. Adherence to antihypertensive medications and cardiovascular morbidity among newly diagnosed hypertensive patients. Circulation. (2009) 120:1598–605. 10.1161/CIRCULATIONAHA.108.830299 19805653

[4] York York Health Economics Consortium & School of Pharmacy University University of London. Evaluation of the Scale, Causes, and Costs of Waste Medicines. The School of Pharmacy University of London (2010).

[5] Nieuwlaat R Wilczynski N Navarro T Hobson N Jeffery R Keepanasseril A. Interventions for enhancing medication adherence. Cochrane Database Syst Rev. (2014) 11:CD000011. 10.1002/14651858.CD000011.pub4 25412402 PMC7263418

[6] Kassavou A Houghton V Edwards S Brimicombe J Sutton S. Development and piloting of a highly tailored digital intervention to support adherence to antihypertensive medication as an adjunct to primary care consultations. BMJ Open. (2019) 9:e024121. 10.1136/bmjopen-2018-024121 30613027 PMC6326276

[7] Kassavou A Sutton S. Reasons for non-adherence to cardiometabolic medications, and acceptability of an interactive voice response intervention in patients with hypertension and type 2 diabetes in primary care: a qualitative study. BMJ Open. (2017) 7:e015597. 10.1136/bmjopen-2016-015597 28801402 PMC5724082

[8] Kassavou A Sutton S. Automated telecommunication interventions to support adherence to cardio-metabolic medication. Health Psychol Rev. (2018) 12:25–42. 10.1080/17437199.2017.1365617 28805162

[9] Kassavou A Houghton V Edwards S Wilson E Brimicombe J Griffin S. Acceptability of the Medication Adherence for Patients Support (MAPS) intervention to improve adherence to patients prescribed multiple medications, as an adjunct to primary care: a qualitative study. J Health Psychol. (2008) 26:168–80. 10.1177/1359105318819051 30565485

[10] Michie S Richardson M Johnston M Abraham C Francis J Hardeman W. The Behavior change technique taxonomy (v1) of 93 hierarchically clustered techniques: Building and international consensus for the reporting of behaviour change interventions. Ann Behav Med. (2013) 46:81–95. 10.1007/s12160-013-9486-6 23512568

[11] Kassavou A Mirzaei V Brimicombe J Edwards S Massou E Prevost AT. A highly tailored text and voice messaging intervention improves medication adherence in patients with either or both hypertension and type 2 diabetes in a UK primary care setting: feasibility randomized controlled trial of clinical effectiveness. JMIR. (2020) 22:e16629. 10.2196/16629 32427113 PMC7267991

[12] Oakley A Strange V Bonell C Allen E Stephenson J. Process evaluation in randomised controlled trials of complex interventions. BMJ. (2006) 332:413–6. 10.1136/bmj.332.7538.413 16484270 PMC1370978

[13] Skivington K Matthews L Simpson SA Craig P Baird J. A new framework for developing and evaluating complex intervention: update of the Medical Research Council guidance. BMJ. (2021) 374:n2061. 10.1136/bmj.n2061 34593508 PMC8482308

[14] Mora P Berkowitz A Contrada RJ Wisnivesky J Horne R Leventhal H. Factor structure and longitudinal invariance of the medication adherence report scale-asthma. Psychol Health. (2011) 26:713–27. 10.1080/08870446.2010.490585 21391132

[15] Barber N. Should we consider non-compliance a medical error? Qual Saf Health Care. (2002) 11:81–4. 10.1136/qhc.11.1.81 12078377 PMC1743570

[16] Ajzen I. The theory of planned behaviour. Organ Behav Hum Decis Process. (1991) 50:179–211. 10.1016/0749-5978(91)90020-T

[17] Bandura A. The Exercise of Control. New York, NY: W. H. Freeman (1997).

[18] Fernandez S Chaplin W Schoenthaler A Ogedegbe G. Revision and validation of the Medication Adherence Self-Efficacy Scale (MASES) in hypertensive African Americans. J Behav Med. (2008) 31:453–62. 10.1007/s10865-008-9170-7 18784996 PMC3763496

[19] Braun V Clarke V. Using thematic analyis in psychology. Qual Res Psychol. (2008) 77–101. 10.1191/1478088706qp063oa32100154

[20] Macharia W Leon G Rowe B Stephenson BJ Haynes RB. An overview of interventions to improve compliance with appointment keeping for medical services. JAMA. (1992) 267:1813–7. 10.1001/jama.1992.03480130129038 1532036

