# Supplementary material for: Process Evaluation of MAPS: A Highly Tailored Digital Intervention to Support Medication Adherence in Primary Care Setting
Source: Front Public Health. 2021 Dec 20;9:806168. doi: 10.3389/fpubh.2021.806168 (PMC8720771; doi:10.3389/fpubh.2021.806168)
Supplement: Supplementary file 1 [file Data_Sheet_1.PDF]

**Supplementary file**

**Table 1**. Examples of the tailored intervention content

| **Determinants** | **Intervention messages** |
|---|---|
| **Advice messages** | |
| Intentional and nonintentional medication adherence, health outcome expectations, self-efficacy, maintenance self-efficacy, social norms | Some people report that they {1} take some of their daily tablets. It is common for people with {HEALTHCONDITION} to {2} their tablets, when they might {3} or they might have to take {4} day. However, taking medications exactly as prescribed is important to keep your {HEALTHOUTCOME} under control. Taking all tablets on time can sometimes be difficult, especially when you {5}, or you might feel {6}. Taking your tablets exactly as prescribed will help you {7} heart attack or stroke in the future.<br>This service will aim to support you to create and maintain a habit for taking and refilling your medications. It will contact you at times and occasions that you have specified at the questionnaire to provide you with advice and support. The messages will include simple reminders to take your tablets on time, and information on how you can manage to take your tablets during challenging circumstances. Thank you for listening. Goodbye |
| Health outcome expectations and maintenance self-efficacy | [name], tablets will help you feel better in the long-term by lowering glucose levels. Please keep taking your medications as prescribed. |
| Intentional and nonintentional nonadherence, and maintenance self-efficacy | [name], continue taking your diabetes type 2 tablets, even when your glucose levels are normal. |
| Social norms and maintenance self-efficacy | [name], if you are planning a change in your routine, ask a friend, a family member, or someone you can rely on, to text/call and remind you about your meds. |
| Social norms and maintenance self-efficacy | [name], tablets taken to treat diabetes type 2 may have side effects. If you have any, contact your GP/pharmacist and get advice. |
| Health outcome expectations and | [name], tablets help you feel better by lowering high blood pressure; whereas stress or worries might increase it. Keep calm and take your tablets. |

| | |
|---|---|
| maintenance self-efficacy | |
| Medication adherence self-efficacy | [name], keep up with your healthy habits and take your 1 tablet doxazosin 2mg at correct times, today. |
| Health outcome expectations | [name], taking your tablets regularly, will reduce your blood pressure below 140/90, if you have hypertension, or below 140/80 if you have diabetes. |
| Intentional and nonintentional nonadherence, medication adherence self-efficacy | [name], for your medications to have an effect, you will need to take them regularly. Please continue taking your medications as prescribed. |
| Medication adherence maintenance self-efficacy | [name], when you are busy, put your high blood pressure & cholesterol meds in a pillbox and take them with meals, or at the bedside table if taken at night. |
| Perceived control | [name], please remember that the effect of your medications wears off after 24 hours. Even if you do not experience any symptoms, it is important to take them every day. |
| **Reminder messages** | |
| Intentional and nonintentional nonadherence, | Hello [name], please do not forget to take your medication today: Ramipril, 1 tablet 2.5mg at 07:00. |
| Intentional and nonintentional nonadherence | Please take your Metformin 2 tablets 500mg at 12:00 to keep up to date. |
| Intentional and nonintentional nonadherence | Hello [name], please do not forget to take Ramipril 1tablet 10mg, Bisoprolol 1tablet 5mg, at 17:30. |
| **Query messages** | |
| Intentional and nonintentional nonadherence | Have you taken all your medications as prescribed today? |

**Interview schedules, practice nurses**

- How many people did you approach about the MAPS trial
- How did they find the invitation to the MAPS trial
- How easy or difficult was it for patients to understand what MAPS trial is about
- How easy or difficult was it to recruit patients to the MAPS trial
- What was easy about recruiting participants to the MAPS trial
- What was difficult about recruiting participants to the MAPS trial
- What were participants' questions about the MAPS trial
- If you were designing the recruitment to the MAPS trial, what would you do differently
- What do you think patients need from an intervention like the MAPS
- What kind of questions do patients ask about the MAPS
- What resources have you used for the MAPS trial
- How useful do you think the MAPS will be in supporting patients to take their medications between their primary care consultations
- Would you recommend the MAPS to other people who take medications for their long-term conditions
- Are there any specific patients who might need the MAPS intervention more than others
- Would you recommend the MAPS as part of the regular care

**Table 2**. Mean changes of theoretical underpinnings of behaviour change

| **Theoretical Determinants** | **Baseline M (95%CI)** | **Follow up M (95%CI)** | **Mean Difference (95% CI)** |
|---|---|---|---|
| Intentional nonadherence | 4.50 (4.23 – 4.77) | 4.82 (4.66 – 4.98) | 0.32 (0.10 – 0.53) |
| Nonintentional nonadherence | 3.96 (3.76 - 4.17) | 4.38 (4.17 – 4.58) | 0.41 (0.15 – 0.66) |
| Health outcome expectations | 1.05 (0.99 – 1.11) | 2.63 (2.35 – 2.90) | 1.57 (1.28 – 1.86) |
| Self-efficacy | 5.91 (5.50 – 6.32) | 6.68 (6.55 – 6.80) | 0.76 (0.33 – 1.19) |
| Self-efficacy, burden of pills | 2.16 (1.93 – 2.39) | 4.36 (4.11 – 4.60) | 2.19 (1.84 – 2.54) |
| Self-efficacy, emotional state | 1.63 (1.41 – 1.84) | 3.86 (3.57 – 4.15) | 2.23 (1.90 – 2.55) |
| Social norms | 6.23 (5.81 – 6.65) | 6.57 (6.35 – 6.79) | 0.33 (-0.11 – 0.79) |

Data are presented as means (M) and 95% Confidence Intervals (CI). Each of the intentional nonadherence, nonintentional nonadherence, health outcome expectations and specific self-efficacy (i.e., burden of pills and emotional state) total scores range from 1-5. Higher scores indicate positive intentional nonadherence, nonintentional nonadherence, health outcome expectations or specific self-efficacy. Generic medication adherence self-efficacy and social norms scores range from 1-7. Higher scores indicate positive self-efficacy or social norms.

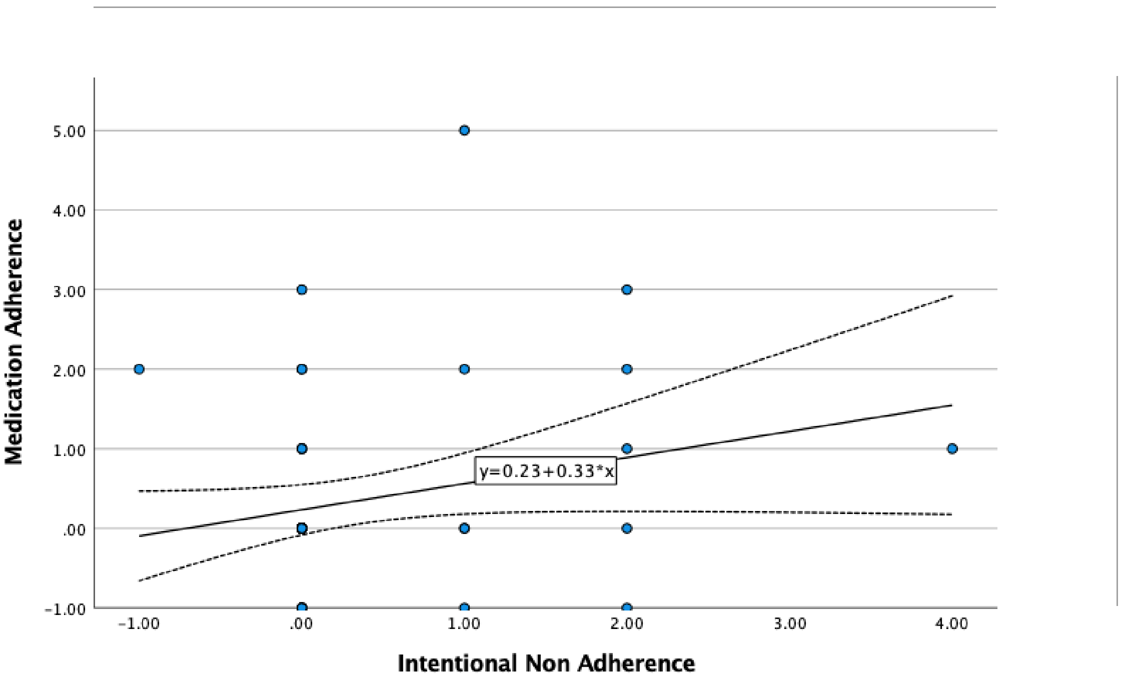


**Figure 1.** The horizontal axis shows changes in intentional non-adherence. Higher numbers indicate improvements in intentional non-adherence. Vertical axis shows the changes in days of medication adherence. Higher numbers indicate improved medication adherence. The line is the fitted regression line showing the association between improvements in intentional non-adherence and medications adherence. The dotted lines are the 95% Confidence Intervals.

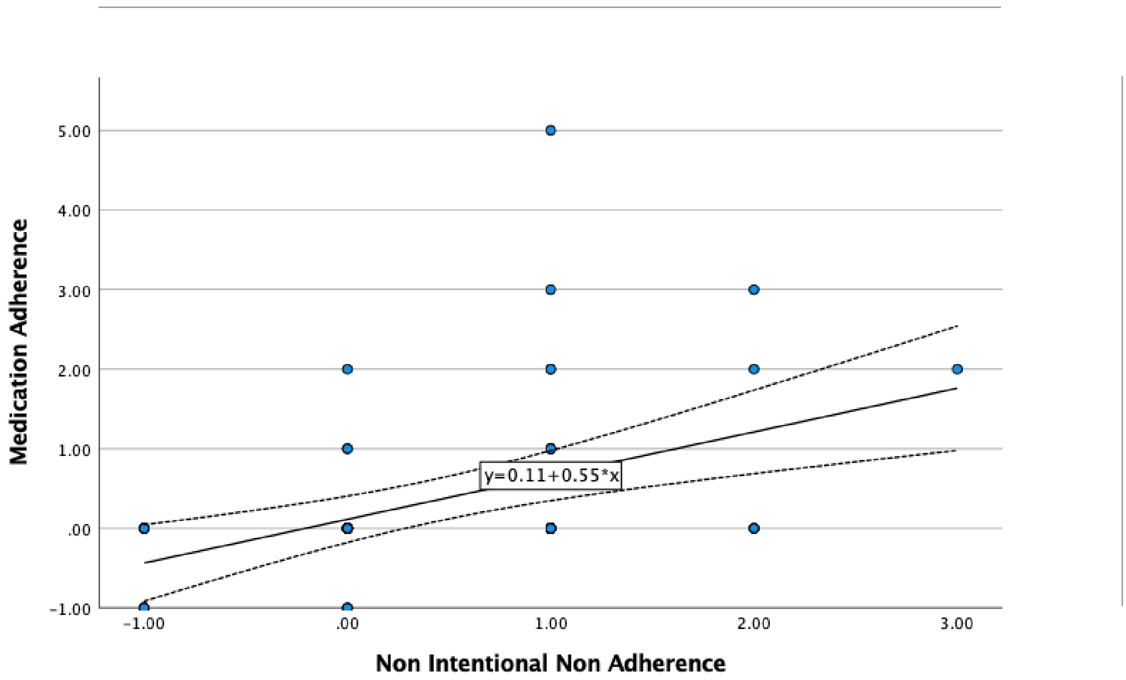


**Figure 2.** The horizontal axis shows changes in non-intentional non-adherence. Higher numbers indicate improvements in non-intentional non-adherence. Vertical axis shows the changes in days of medication adherence. Higher numbers indicate improved medication adherence. The line is the fitted regression line showing the association between improvements in non-intentional non-adherence and medications adherence. The dotted lines are the 95% Confidence Intervals.

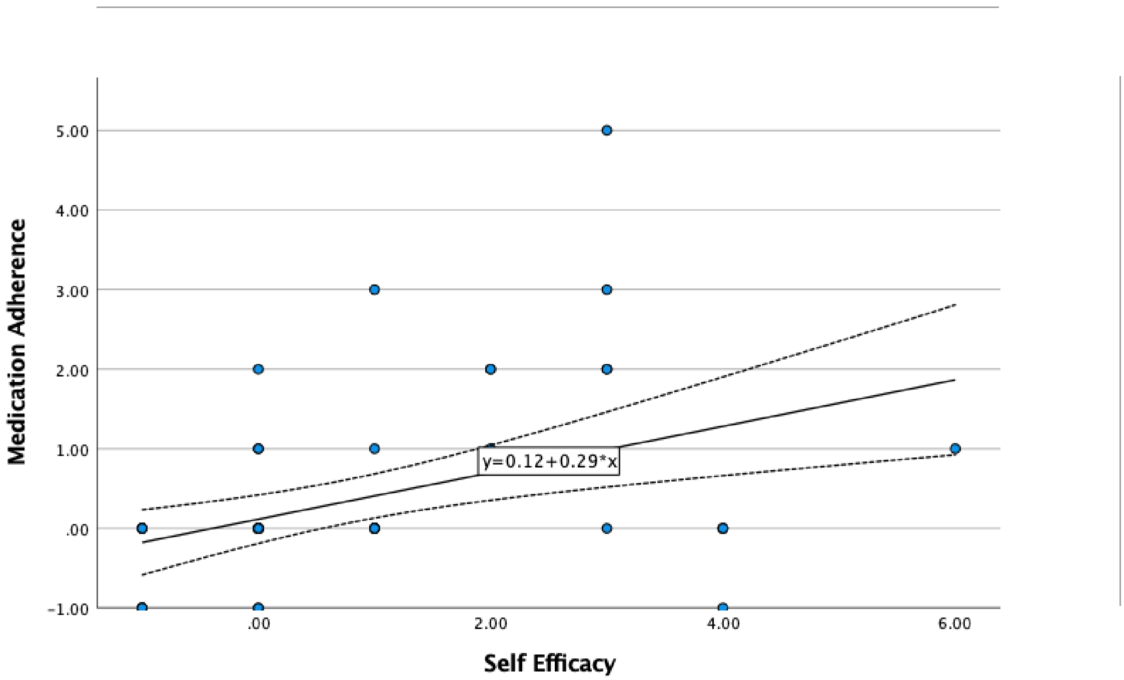


**Figure 3.** The horizontal axis shows changes in self-efficacy. Higher numbers indicate improvements in medication adherence self-efficacy. Vertical axis shows the changes in days of medication adherence. Higher numbers indicate improved medication adherence. The line is the fitted regression line showing the association between improvements in self-efficacy and medications adherence. The dotted lines are the 95% Confidence Intervals.

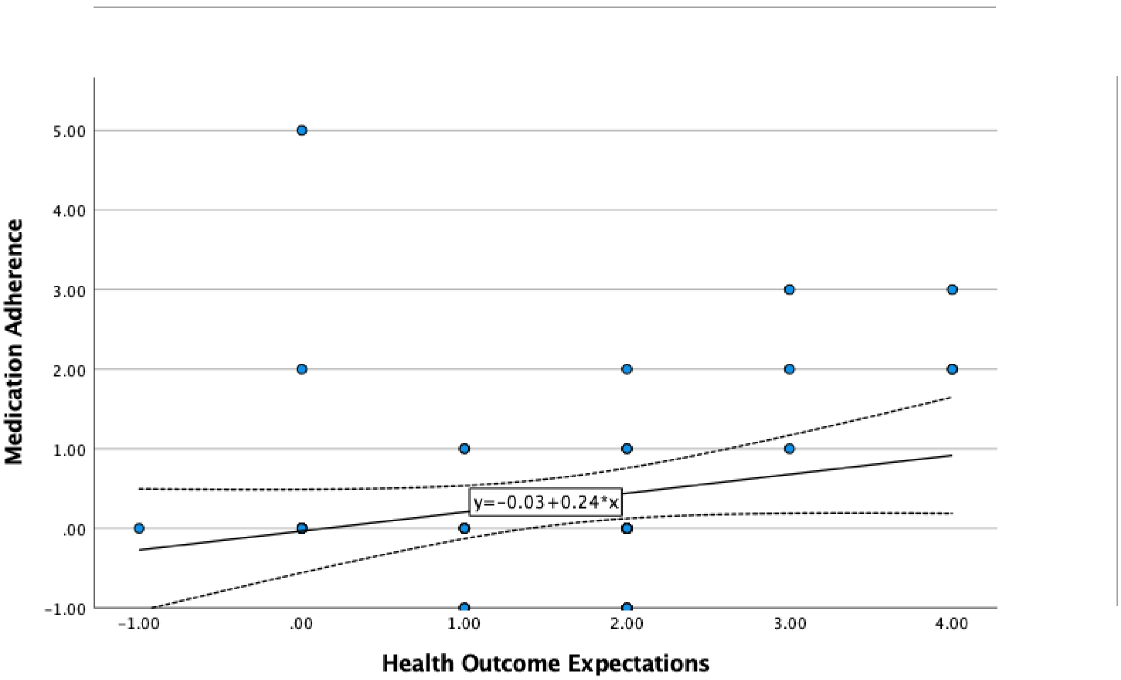


**Figure 4.** The horizontal axis shows changes in health outcome expectations. Higher numbers indicate improvements in health outcome expectations. Vertical axis shows the changes in days of medication adherence. Higher numbers indicate improved medication adherence. The line is the fitted regression line showing the association between improvements in health outcome expectations and medications adherence. The dotted lines are the 95% Confidence Intervals.

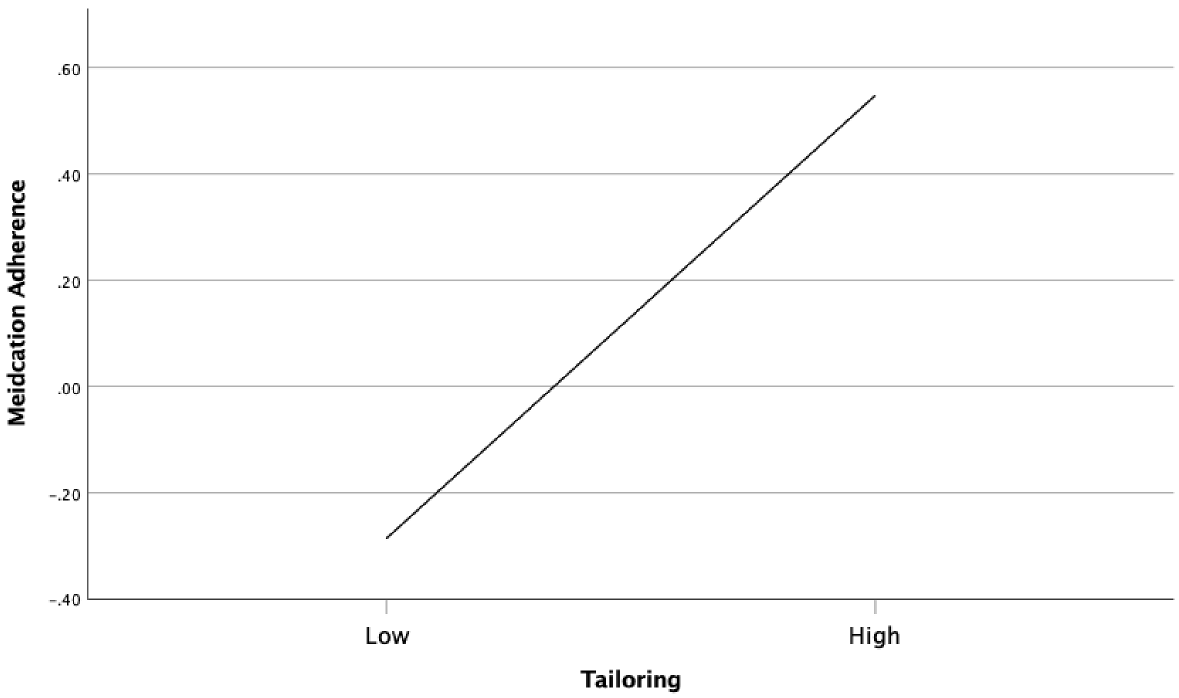


**Figure 5.** The horizontal axis shows tailoring. Low indicates less tailoring and high indicate more tailoring advice. Vertical axis shows the changes in days of medication adherence. Higher numbers indicate improved medication adherence. The line is the fitted regression line showing the association between intervention tailoring and changes in medication adherence.

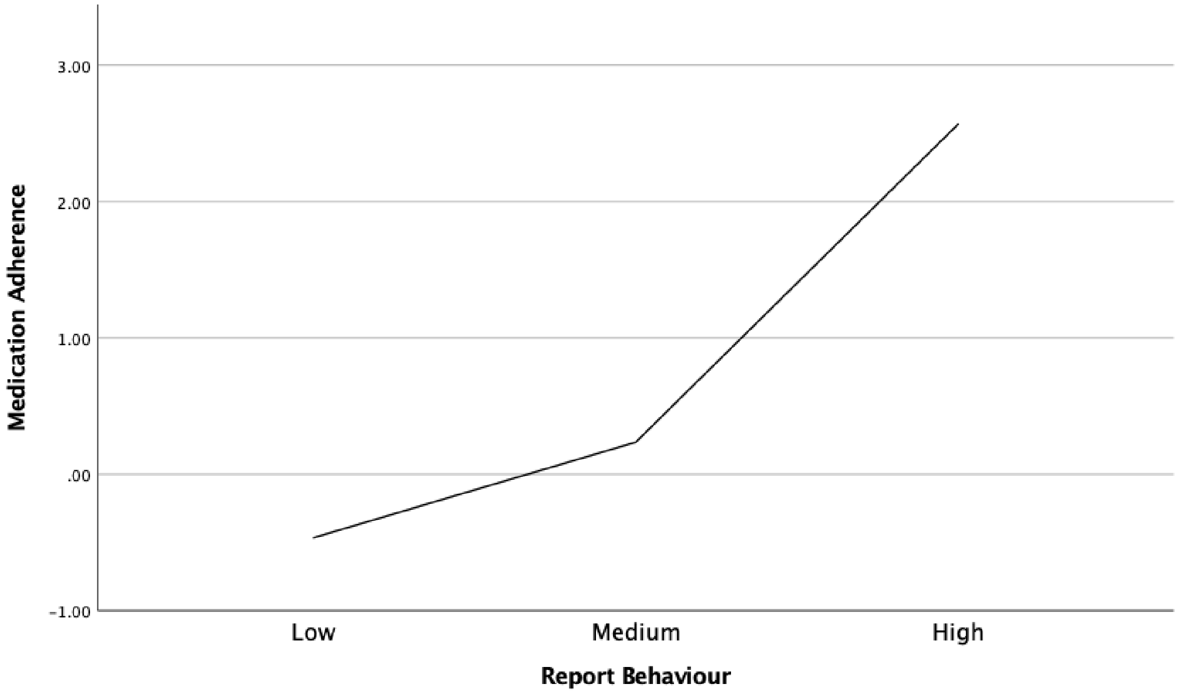


**Figure 6.** The horizontal axis shows report on behaviour. Low indicates less than half of the responses confirmed adherence, medium when at least half of the responses indicated adherence and high when all responses indicated adherence. Vertical axis shows the changes in days of medication adherence. Higher numbers indicate improved medication adherence. The line is the fitted regression line showing the association between reports on behaviour and changes in medication adherence.
